# On a New Property of the Hydrocyanic Acid Taken Internally

**Published:** 1821-10

**Authors:** R. Macleod

**Affiliations:** one of the Physicians to the Westminster General Dispensary, to the Royal Infirmary for Children, and to the Scottish Hospital.


*On a new Property of the Hydrocyanic Acid taken internally.* By R. Macleod, M.D. one of the Physicians to the Westminster General Dispensary, to the Royal Infirmary for Children, and to the Scottish Hospital.

AT the time the hydrocyanic acid was first recommended as a medicine in this country, I was induced to try it on rather an extensive scale at the Westminster General Dispensary. Following the directions of Magendie and Dr. Granville, I administered it in pectoral complaints, chiefly phthisis in its various stages, and the chronic bronchitis of elderly persons. The result of sixty such cases satisfied me that this medicine had either been extolled above its deserts, or that its real powers were not rightly understood; and I discontinued its exhibition, partly because I was unable to discover any certain indication which it was capable of fulfilling, and partly because the general relief afforded by it, in the cases referred to, was inferior to what was produced by more common remedies. I must remark, however, that the apprehension of deleterious effects entertained by some, and noticed by several who have written on the subject, appear to me ill-founded: at least, out of above one hundred cases, in which I have given, or seen others give, the hydrocyanic acid, it never has produced serious inconvenience, except once, and even then the effects were not alarming.

In complaints of a spasmodic nature, such as hooping-cough, I believe this acid to be of use; but the only class of cases in which I feel any real confidence in its exhibition, is dyspepsia attended with much pain in the stomach, or anomalous feelings about the chest and heart. Indeed, I have known it afford very decided relief in structural affections of this organ; a circumstance first forced upon my attention by seeing it prescribed to a patient under my care at the Westminster Dispensary, in which instance the freedom from suffering was complete and permanent,—that is, it lasted till the patient's death, which took place two days after its exhibition. On examining the heart, with my friend Dr. Richard Harrison, we found it enlarged and flabby, with thickening of the central valves; the coronary arteries containing a considerable quantity of air.

The more particular object, however, of the present short communication is to notice an effect of this medicine, which, so far as I know, has not been remarked by any of the writers on this subject,—viz. ulceration of the gums, with salivation. Of this I have seen three instances: in the first it was slight, and, not being aware of this peculiar property of the acid, I regarded it as proceeding from some other cause. The second instance occurred in a man named Smith, residing in Exeter-street; and, my attention being excited by the occurrence of the same effect in two individuals while using the acid, I was resolved to ascertain how far it was to be regarded as the cause. Smith had taken, during a fortnight, from six to eight table-spoonsful daily of the following mixture:—℞. Acid. hydrocyanei, m. x.; Aquæ distillatæ, f. ℥viij. M. At the end of this time, the ulceration of the gums, which had been gradually coming on, was considerable, and attended with some salivation. The prussic acid was discontinued, an alum wash was ordered, and the gums gradually got well. About three months after this, his mouth having been well for some weeks, I again prescribed the same mixture, and the same effects were again produced.

A young woman in Phœnix-street, a patient at the Westminster General Dispensary, had the above mixture ordered, of which she took three table-spoonsful three times a-day for a fortnight; when, on visiting her after a lapse of several days, I found the gums affected with severe ulceration, extending to the inside of the left cheek, accompanied by salivation and considerable swelling. The ulcerations were very difficult to heal, and the soreness of the mouth was altogether exceedingly distressing. After it had been well for about three months, I again ordered a mixture containing the same quantity of acid as before. The dose was rapidly increased to four table-spoonsful four times a-day, and at the end of a week the mouth had again become affected. The gums were swelled, inflamed, and slightly ulcerated; but got well more speedily than before.

I conceive that the above cases warrant the conclusion that hydrocyanic acid has the property of inducing, in some constitutions, an effect upon the mouth analogous to that resulting from the use of mercury. But there seems to be this difference, —that the ulceration of the gums is greater in proportion to the degree of salivation, and I believe the inconvenience to be more difficult of cure. The occurrence of this effect cannot, I suspect, be regarded as favourable. In the former of the two instances related, it was exhibited for a pectoral affection, as recommended by Dr. Granville: in the latter, it was given for disease of the digestive organs, as recommended by Dr. Elliotson: in neither case did the medicine prove a remedy.

---

*Note to the above Paper.* By the Editor.

Nothing, in our opinion, enables us more readily to form a just valuation of the power of new medicines, than the candid recital of failures in cases where those medicines have been employed with an expectation of success. That Dr. Macleod, in his extensive field of practice at the Westminster General Dispensary, should have oftener failed than succeeded in obtaining relief from the hydrocyanic acid in cases of pectoral diseases, can no more be construed into a direct acknowledgment of the inutility of that medicine, than a more favourable report of its virtues is to be looked upon as a declaration of its infallibility. Dr. Macleod himself knows that, in one, two, or three instances at least, the hydrocyanic acid, prescribed by himself or by the Editor conjointly, produced all the benefit that was expected from that medicine. In the treatment of whooping-cough, we hold this agent to be of incalculable utility; and we have had occasion, since we first asserted the fact, and again repeated it in the second edition of our work upon Prussic Acid, to call the attention of Dr. Macleod himself to the advantage derived from its use, in numerous cases of pertussis, at the Royal Infirmary for Children, of which Dr. Macleod is one of the very zealous medical officers. The register of the central station of that extensive charity will prove that, of upwards of one hundred cases of pertussis admitted within the last seven or eight months, not three of them have ended fatally; the remainder having recovered, in the course of a short time, under the use of the hydrocyanic mixture, with the exception of about a dozen cases. In the instance of another officer of the same Institution, who had suffered under a most alarming attack of pneumonia, the cough, which remained behind after his recovery, yielded only to the prussic acid, and that in a few days.

That the evidence resulting from any one set of experiments, respecting the virtues of a particular medicine, is not sufficient to form an opinion, can be proved, by opposing to the report of Dr. Macleod's candid statement, many similarly candid relations of cases, taken from various writers, which were crowned with success. But it is a singular coincidence, that, at the time when Dr. Macleod supplied me, at my request, with the foregoing paper, I should have accidentally met with another report of Dispensary practice, relative to the use of prussic acid in pulmonic diseases, by Dr. Nancrede, of Philadelphia, from which I must crave permission to quote the three following cases.

"Case I.—John C. aged about fifty-three years, of a strong and muscular appearance, working in a brick-yard, had been troubled for about two months, in August 1819, with continual pain in the chest, dyspnœa, and cough, accompanied with some expectoration of a doubtful nature. The pain in the chest induced me to direct the loss of about fourteen ounces of blood, and immediately after he began the use of the acid in daily doses of four drops, increasing two drops every day, until he had taken twenty-four in a day. He now informed me that his cough was somewhat better, and that his sleep, which had been very much disturbed by unpleasant dreams, and otherwise agitated, was more tranquil. The dose was continued thus for a few days, and then increased four drops daily, until the patient took daily thirty-six drops. The benefit derived had increased; his cough was better, and his sleep continued composed. The medicine was now at an end, and this case terminated here. It is, however, proper to mention, that I have seen him a month ago, still affected with slight cough, but which is considerably diminished. He is now enabled to pursue his work. I conclude, therefore, that in this case the prussic acid has been of some use.

"Case II.—In November last, Robert N—, a patient of the Dispensary, aged about thirty-six years, of a slender form, and by trade a carpenter, applied for medical assistance. He complained of pain in the chest, great difficulty in breathing, or (as he expressed it) a shortness of breath, a dry cough, no expectoration, and occasional chills and night-sweats. His pulse was tense and frequent. These symptoms had been gradually increasing for about six months, during which time the patient had lost considerable flesh. After taking, for a few nights, the ipecacuanha and opium combined, he began the use of the acid. At first he took it in very small doses, owing to the impression I was then under that its effects would be very powerful; but the second prescription (November 28th,) contained thirty drops in four ounces of water, edulcorated with gum arabic and sugar. The patient was directed to take a table-spoonful of the mixture three times each day; and he soon felt some amelioration in his feelings.

"*December 2d.*—The mixture being out, a new one, containing ʒij prussic acid in the same vehicle, and accompanied with the same injunctions as to its doses, was directed. It was followed by increased benefit.

"*Dec. 7th.*—One drachm of the acid in three ounces of water, edulcorated with gum arabic and sugar, which the patient is to take in table-spoonfuls every third hour. Robert states that his cough is considerably diminished.

"*Dec. 13th.*—The same prescription is renewed, with manifest advantage.

"*Dec. 18th.*—One drachm and a half in six ounces of water is prescribed, to be used in the same doses, and at the same intervals. The patient continues improving. At no period since its first exhibition has he mentioned either nausea or head-ache.

"Robert continued under the operation of the acid until the 20th of January, and then used, as a substitute and general tonic, the cold infusion of bark. His cough has nearly left him; and his appetite, appearance, and general feelings, are about the same as previous to the invasion of the disease. He is discharged from the care of the institution, and is now pursuing his trade.

"Case III.—Mary P—, aged about twenty-two years, had been affected for about three weeks with the usual symptoms of incipient phthisis, and pronounced to be affected with that disease by a respectable physician, who had visited her previous to my attendance. When I first called on her, on the 11th of February, I found the patient confined to her bed with continual fever, and a small, quick, and tense pulse. She had already used, in vain, several pectoral mixtures, and took, according to my directions, a fresh one containing balsam tolu; but without deriving from either any relief whatever. Accordingly, very soon after my attendance commenced, the prussic acid was administered, in doses of one drop to the ounce of water; which doses were very soon increased. She is now taking it in doses of sixteen drops to four ounces of water,—a table-spoonful every two hours. Without exhausting your patience, gentlemen, with details which, by their repetitions, may be tedious and uninteresting, I will merely state that my patient is rapidly improving; so much so, that this day, the 11th of March, she informs me that she has not coughed more than twice or three times in the course of the last twenty-four hours. She is now sitting up, and has already acquired some flesh, and a slow and rational pulse."—(*Philadelphia Journal, May* 1821.)

A curious fact, however, particularly if future observations should confirm it, for the knowledge of which the profession stand indebted to Dr. Macleod, is the property which the hydrocyanic acid seems to possess of exciting salivation in some cases. My attention having been called to the circumstance by that gentleman some weeks ago, I had an opportunity of explaining the (till then unaccountable) occurrence of salivation, in my practice, in the cases of two children affected with the whooping-cough, who were taking the medicine in question. In those two instances, I had endeavoured to account for the increased flow of saliva, and the attending ulceration of the buccal mucous membrane, by supposing that the mother had administered mercurial powders previously to my prescribing any medicine for the patients.

The fact being now asserted, it rests with the profession to investigate it, and to ascertain how far, and under what circumstances, it takes place; and we shall feel indebted for any communication which those of the profession who have the advancement of science at heart will be kind enough to address to us on the subject.—A. B. G.

